# RGB-D SLAM Combining Visual Odometry and Extended Information Filter

**DOI:** 10.3390/s150818742

**Published:** 2015-07-30

**Authors:** Heng Zhang, Yanli Liu, Jindong Tan, Naixue Xiong

**Affiliations:** 1 School of Information Engineering, East China Jiaotong University, Nanchang 330013, China; E-Mail: hzhang69@utk.edu; 2 Department of Mechanical, Aerospace and Biomedical Engineering, University of Tennessee, Knoxville, TN 37996, USA; E-Mail: jdtan122@utk.edu; 3 School of Computer Science, Colorado Technical University, Colorado Springs, CO 80907, USA; E-Mail: dnxiong@ieee.org

**Keywords:** SLAM, visual odometry, extended information filter, binary descriptor

## Abstract

In this paper, we present a novel RGB-D SLAM system based on visual odometry and an extended information filter, which does not require any other sensors or odometry. In contrast to the graph optimization approaches, this is more suitable for online applications. A visual dead reckoning algorithm based on visual residuals is devised, which is used to estimate motion control input. In addition, we use a novel descriptor called binary robust appearance and normals descriptor (BRAND) to extract features from the RGB-D frame and use them as landmarks. Furthermore, considering both the 3D positions and the BRAND descriptors of the landmarks, our observation model avoids explicit data association between the observations and the map by marginalizing the observation likelihood over all possible associations. Experimental validation is provided, which compares the proposed RGB-D SLAM algorithm with just RGB-D visual odometry and a graph-based RGB-D SLAM algorithm using the publicly-available RGB-D dataset. The results of the experiments demonstrate that our system is quicker than the graph-based RGB-D SLAM algorithm.

## 1. Introduction

Self-localization is widely recognized as one of the most basic problems for an autonomous robot with respect to navigation. This task can be performed pretty well when the environment is known *a priori*, but when a map of the environment is not available beforehand, robot localization becomes very difficult. This may be due to a lack of information of the environment that the robot moves in or to the excessive cost of manually building a map on purpose. In these cases, the robot must simultaneously build a map of the environment and localize itself within it. This problem, known as simultaneous localization and map building (SLAM), has been extensively studied over the last two decades. The solutions to the SLAM problem presented so far differ mainly for the environment description adopted and for the estimation technique employed. There are two main estimation forms: filter-based SLAM and graph-based SLAM.

Filter-based SLAM involves estimating the posterior by means of Bayes’ rule [1]:

(1) $$p({\text{ξ}}_{t},m|z_{1:t},u_{1:t})$$

where ${\text{ξ}}_{t}$ is the pose of the robot at time *t*, *m* is the map, $z_{1:t}$ is the observation sequence and $u_{1:t}$ is the odometry information (or motion control input). Filter-based SLAM is also called online SLAM, since it is incremental; past measurements and controls are discarded once they have been processed. According to different ways of addressing the posterior probability, there are many filter-based methods like the extended Kalman filter (EKF) method [2], the extended information filter (EIF) method [3], the particle filter (PF) method [4], *etc*.

Instead of estimating only current pose ${\text{ξ}}_{t}$ in filter-based SLAM, the graph-based SLAM estimates a complete trajectory ${\text{ξ}}_{1:t}$ and map *m* by all observed information. The method is considered time-consuming and cannot satisfy real-time requirements. However, by means of efficient solving methods, the graph-based SLAM has received more attention [5,6,7].

The initial studies on the SLAM problem focused on two-dimensional environments, so they were usually applied to mobile robots. Recently, a variety of 3D SLAM algorithms have supported 6-DOF (degree-of-freedom) pose optimization [8]; therefore, the SLAM technique is employed in various platforms, such as quadrotors [9], underwater robots [10], *etc*. In the early 3D SLAM studies, expensive sensors, like 2D and 3D-LRFs (laser range finders), were mainly used. However, recently, with the advent of inexpensive Kinect-style sensors [11], which are called RGB-D (red-green-blue depth) cameras, since they give the color image and the depth data concurrently, the robotics and computer vision communities have focused on 3D SLAM techniques using the RGB-D camera data; we call these techniques RGB-D SLAM.

The current RGB-D SLAM techniques, which are reviewed in detail in the following section, are mostly based on graph-based SLAM. They need loop detection and loop correction to refine the graph, and sometimes, they are not real time. For most RGB-D SLAM systems, there are three major problems. (1) The hyper-high dimensional problem: In two-dimensional space, the pose is represented as ${\text{ξ}}_{t}={\left(x_{t},y_{t},\theta_{t}\right)}^{T}$, and the environmental map is denoted as $\text{Θ}=\{\theta_{1},\theta_{2},\cdots ,\theta_{N}\}$, which contains *N* characteristics, where $\theta_{i}=\left(x^{i},y^{i}\right)$. The dimension of the state vector is 2N+3. For the three-dimensional space, the pose is represented as ${\text{ξ}}_{t}={\left(x_{t},y_{t},z_{t},\alpha_{t},\beta_{t},\gamma_{t}\right)}^{T}$, and the dimension of the state vector is 3N+6. Since the number of features in the actual environment may reach tens of thousands, the SLAM problem is a hyper-high dimensional problem; (2) The data association problem: This problem means that the extracted feature is judged as to whether it is a new or pre-existing feature. Assume at time step *t*, *m* extracted features match *n* features in the map with computation complexity $O(n^{m})$ irrespective of the independence between features. The time complexity is very high for a real-time environment; (3) The selection and design of visual odometry: Frame-to-frame alignment based on feature matching should not be selected to avoid over-estimation. It is caused by re-applying the feature measurements that are used both as motion information and as measurement information in the SLAM process.

In this paper, we propose a new RGB-D SLAM method based on visual odometry (VO) and the extended information filter (EIF), referred to as VO-EIF RGB-D SLAM. As with current graph-based RGB-D SLAM algorithms, our filter-based RGB-D SLAM in this paper does not depend on other sensors (such as gyroscope, encoder, *etc*.). Our contribution consists of providing an appropriate observation model and motion model for the SLAM for a robot. More concretely, this paper has the following contributions: (1) we adopt the method based on the extended information filter to decrease the dimensions for a high-dimensional state space; (2) inspired by the related works [12,13,14], we employ the binary feature descriptor for feature matching to reduce the complexity effectively; (3) we build an RGB-D feature observation model that combines the 3D positions and the binary descriptors of the landmarks and that avoids explicit data association between the observations and map; and (4) we devise a visual dead reckoning algorithm based on visual residuals, which is used to estimate motion control input, to avoid over-estimation. Moreover, this is more robust and accurate than feature-based visual odometry methods.

The rest of the paper is organized as follows: Section 2 refers to the related work. Section 3 provides the principle of the extended information filter SLAM. Section 4 describes the binary robust appearance and normals descriptor (BRAND) descriptor. The RGB-D feature observation model and the motion model in this work are introduced in Section 5 and Section 6, respectively. Section 7 shows the experimental results, and Section 8 sets out the conclusions and presents lines for future work.

## 2. Related Research

Currently, most robot SLAM is carried out with the sensor, which provides a 2D scene. The main reason is that acquiring high-quality 3D data is very expensive. However, with the advent of the low-cost Microsoft Kinect sensor, there has been great interest in capturing and reconstructing 3D environments using a movable RGB-D sensor [7,15,16]. It provides dense, high-resolution depth information at a low price and small size.

Fioraio *et al.* [17] developed a SLAM application using Kinect. They used the bundle-adjustment framework to ingrate ICP (iterative closest point) [18] with visual feature matches. In their research, the graph is optimized using a $g^{2}o$ (general graph optimization) framework [5] to obtain global alignment. They adopted the ICP algorithm [18] for pairwise alignment between sequential frames and recovering the rigid transformation between point clouds. The alignment accuracy of ICP significantly depends on the scene content.Po-Chang *et al.* [19] use color feature descriptors to improve depth data correspondences. Lee *et al.* [20] proposed an RGB-D SLAM method that handles low dynamic situations using a pose-graph structure, in which nodes are grouped based on their covariance values. Any false constraints are pruned based on an error metric related to the node groups.

Henry *et al.* [15,16] studied highly efficient pose graph optimization, such as TORO (tree-based network optimizer) in 2010. In 2012, Henry *et al.* [16] improved this algorithm. They combined FAST (features from accelerated segment test) and Calonder descriptors [21] to estimate pose, utilized the RE-RANSAC (re-projection error random sample consensus) method for frame-to-frame alignment and incorporated ICP constraints into SBA [22] (sparse bundle adjustment) for global optimization. The core of their algorithm is RGB-D ICP, a novel ICP variant that makes use of the rich information included in RGB-D data. In 2013, Henry *et al.* [23] presented patch volumes to create globally-consistent maps. The approach combines GPU-accelerated volumetric representations with global consistency, which shows the great effect for indoor map building.

Audras *et al.* [24] presented ab RGB-D SLAM methodology that is very efficient for a complex indoor environment. In the algorithm, the trajectory estimation is integrated into a single global process, which does not rely on intermediate-level features. Moreover, using the accurate pose measurement with the localization techniques, a compact photometric model of the environment is acquired. In [25], the rigid body motion of a handheld RGB-D camera is estimated by an energy-based approach. They combined visual odometry technology with an RGB-D sensor for autonomous flight experimental analysis. The experimental system is able to plan a complex 3D path in a cluttered environment. The work in [24,25] do not extract sparse features and warp the pixel for one frame to another using a depth map and a photometric error minimization method for frame-to-frame alignment. The work in [26] presents a new dense visual odometry system, in which the geometric error is parameterized by the inverse depth instead of the depth, as used in most VO systems.

The work in [27] puts forward a novel GPU implementation based on an RGB-D visual odometry algorithm. They used a 6-DOF camera odometry estimation methods to track and integrate RGB color information into the KinectFusion [28] reconstruction process to allow a high-quality map. The experiment shows that there is no need for the use of keyframes, and the method results in real-time colored volumetric surface reconstructions. Many RGB-D SLAM techniques are limited to office-type and geometrically-structured environments. Hu *et al.* [29] proposed a switching-based algorithm that heuristically choose between RGB-D bundle adjustment and RGB-D bundle adjustment-based local map building. RGB-D SLAM maps are created by applying sparse bundle adjustment on an included two-step re-projection RANSAC and ICP approach. By a heuristic switching algorithm, they dealt with various failure modes associated with RGB-D-BA (RGB-D bundle adjustment). The map connection strategy significantly reduces the computational cost, and the algorithm has great potential to be applied in a larger scale environment.

Similar to [15,16], Endres *et al.* [30] used the ICP algorithm only when there were few or no matching keypoints in order to reduce the time complexity. They used the $g^{2}o$ framework [5] to optimize the 3D pose graph and created a stereo 3D map for robot localization, navigation and path planning. The work in [31] uses RGB-D data to provide a complete benchmark for evaluating visual SLAM and odometry systems and proposes two evaluation metrics and automatic evaluation tools.

Kerl *et al.* [32] proposed a dense visual SLAM method for RGB-D cameras and an entropy-based similarity measure for keyframe selection and loop closure detection. In contrast to sparse, feature-based methods [15,30], the approach significantly decreases the drift and is real time. Compared to the work by Tykkala *et al.* [33], the keyframe and pose graph optimization are obtained simultaneously without manual intervention. Since traditional loop closures have a high time complexity, the work in [34] presents a novel SLAM system that takes advantage of non-rigid map deformations for map correction during loop closures.

Felix Endres *et al.* [35] extracted keypoints from the color images and used the depth images to localize them in 3D. RANSAC is used to estimate the transformations between associated keypoints and to optimize the pose graph using nonlinear optimization. In contrast to other RGB-D SLAM system, they performed a detailed experimental evaluation on benchmark dataset [31] and discussed many parameters, such as the choice of the feature descriptor, the number of visual features, *etc*. The system is robust for scenarios such as fast camera motions and feature-poor environments.

Most RGB-D SLAM algorithms [15,24,25,27,36,37,38] combine texture with geometric features to deal with the problem and regard the scene as a set of points. The work in [39] exploits the structure of the scene and incorporates both point and plane features to implement the SLAM algorithm. The algorithm explains how to find point and plane correspondences using camera motion prediction and uses both points and planes to relocate and bundle adjustment, aiming at refining camera pose estimates.

**Figure 1:**
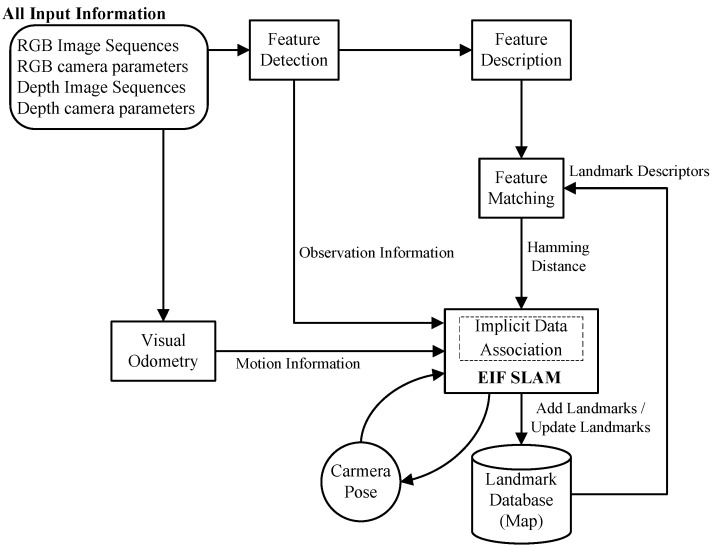
Flow diagram of the visual odometry-extended information filter (VO-EIF) RGB-D SLAM system. Through the data association test, the landmark is added to the landmark descriptor database for the feature matching. The core of the system is the EIF SLAM. Section 3 introduces the extended information filter approach. The binary descriptor is demonstrated in Section 4. The observation model and the motion model are presented in Section 5 and Section 6, respectively.

We put forward the RGB-D SLAM algorithm based on the traditional filter-based SLAM algorithm in the paper. Firstly, we apply the unsupervised learning algorithm without human intervention [40] to correct depth distortion. Then, the BRISK (Binary Robust Invariant Scalable Keypoints) keypoint detector [13] is adopted to extract feature points, and next, we use the BRAND [12] descriptor to increase the credibility of data association in SLAM. Finally, We develop the RGB-D SLAM system integrating EIF SLAM with dense visual odometry (DVO). The flow diagram of the algorithm is illustrated in Figure 1. The descriptor and the VO-EIF RGB-D SLAM algorithm will be described in detail in the following sections.

## 3. Extended Information Filter Approach to SLAM

In the SLAM algorithm, the state vector $s_{t}$ consists of RGB-D camera pose ${\text{ξ}}_{t}$ and the set of *n* map landmarks, *i.e*., $s_{t}={\left[{\text{ξ}}_{t},p_{m}^{1},p_{m}^{2},\cdots p_{m}^{n}\right]}^{T}$, where $p_{m}^{j}$ is the 3D position coordinate of the *j*-th landmark in the world coordinate system at time step *t*. We use a first-order linearization of the motion and measurement models. Assume posterior $p(s_{t}|z^{t},u^{t})$ obeys a Gaussian probability distribution, traditionally parameterized by the mean $\mu_{t}$ and the covariance matrix ${\text{Σ}}_{t}$.

(2) $$\begin{array}{ccc} & & p\left(s_{t}|z^{t},u^{t}\right)=N\left(\mu_{t},{\text{Σ}}_{t}\right)\\ & \propto & \exp\left\{-\frac{1}{2}{\left({\text{ξ}}_{t}-\mu_{t}\right)}^{T}{\text{Σ}}_{t}^{-1}\left({\text{ξ}}_{t}-\mu_{t}\right)\right\}\\ & = & \exp\left\{-\frac{1}{2}\left({\text{ξ}}_{t}^{T}{\text{Σ}}_{t}^{-1}{\text{ξ}}_{t}-2\mu_{t}^{T}{\text{Σ}}_{t}^{-1}{\text{ξ}}_{t}+\mu_{t}^{T}{\text{Σ}}_{t}^{-1}\mu_{t}\right)\right\}\\ & \propto & \exp\left\{-\frac{1}{2}{\text{ξ}}_{t}^{T}{\text{Σ}}_{t}^{-1}{\text{ξ}}_{t}+\mu_{t}^{T}{\text{Σ}}_{t}^{-1}{\text{ξ}}_{t}\right\}\\ & = & \exp\left\{-\frac{1}{2}{\text{ξ}}_{t}^{T}{\text{Λ}}_{t}{\text{ξ}}_{t}+\eta_{t}^{T}{\text{ξ}}_{t}\right\}\\ & \propto & N^{-1}\left(\eta_{t},{\text{Λ}}_{t}\right) \end{array}$$

where $z^{t}=\left\{z_{0},z_{1},\cdots ,z_{t}\right\}$ denotes the history of observational data, $z_{t}=\left\{z_{t}^{i}|i=1,2,\cdots ,N_{t}\right\}$ denotes the observational data of the RGB-D camera and $z_{t}^{i}$ denotes the observational data of the i-th landmark at time step *t*. $u^{t}=\left\{u_{1},\cdots ,u_{t}\right\}$ is the history of motion control inputs;$u_{t}={\left(\text{Δ}x_{t},\text{Δ}y_{t},\text{Δ}z_{t},\text{Δ}\alpha_{t},\text{Δ}\beta_{t},\text{Δ}\gamma_{t}\right)}^{T}$ is the motion control inputs of the RGB-D camera at time step *t*. Gaussian probability distribution Equation (2) is parameterized by the information vector $\eta_{t}$ and the information matrix ${\text{Λ}}_{t}$.

(3) $$\begin{array}{ccc} {\text{Λ}}_{t} & = & {\text{Σ}}_{t}^{-1} \end{array}$$

(4) $$\begin{array}{ccc} \eta_{t} & = & {\text{Λ}}_{t}\mu_{t}={\text{Σ}}_{t}^{-1}\mu_{t} \end{array}$$

Extended information filtering is similar to the extended Kalman filter. The algorithm is divided into two phases: measurement update and state prediction [3].

Measurement update: The key of landmark observation is to reduce the uncertainty in the estimates for the camera pose and the map. The general measurement model Equation (5) is a nonlinear state function with added white Gaussian noise, $v_{t}∼N\left(0,R\right)$. Equation (6) is the first-order linearization related to the mean of the robot pose and observed features with the Jacobian.

(5) $$\begin{array}{ccc} z_{t} & = & h\left(s_{t}\right)+v_{t} \end{array}$$

(6) $$\begin{array}{ccc} & \approx & h\left({\bar{\mu }}_{t}\right)+H\left(s_{t}-{\bar{\mu }}_{t}\right)+v_{t} \end{array}$$

Use $p\left(s_{t}|z^{t-1},u^{t}\right)=N^{-1}\left({\bar{\eta }}_{t},{\bar{\text{Λ}}}_{t}\right)$ to update the current distribution and applying Bayes’ rule to infer a new observation.

(7) $$p\left(s_{t}|z^{t},u^{t}\right)\propto p\left(z_{t}|s_{t}\right)p\left(s_{t}|z^{t-1},u^{t}\right)$$

**Figure 2:**
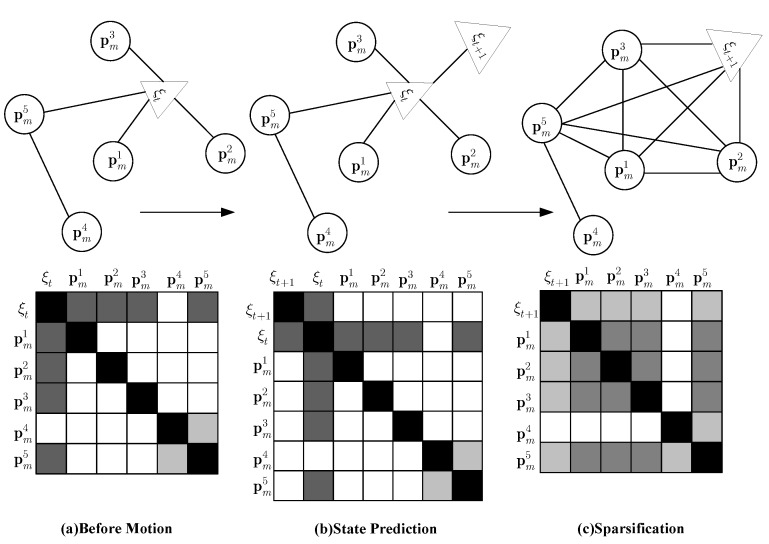
A graphical explanation of EIF’s methodology in the information matrix. A circle indicates the position of a feature; a triangle indicates the camera pose. Darker shades in the matrix imply stronger relevance; white indicates no relevance. (**a**) $m^{+}=\left\{p_{m}^{1},p_{m}^{2},p_{m}^{3},p_{m}^{5}\right\}$ represents features associated with the camera pose; $m^{-}=\left\{p_{m}^{4}\right\}$ represents features irrelevant to the camera pose; (**b**) new camera pose ${\text{ξ}}_{t+1}$ is added to the state vector ${\hat{s}}_{t+1}={\left[{\text{ξ}}_{t},{\text{ξ}}_{t+1},M\right]}^{T}$; (**c**) marginalized distribution with the old pose ${\text{ξ}}_{t}$. The constraints between ${\text{ξ}}_{t}$ and each map element in $m^{+}$ are eliminated. New constraints between ${\text{ξ}}_{t+1}$ and $m^{+}$ are created. We see from the shading that many constraints between features are weakened.

State prediction: The prediction stage predicts the distribution over the new camera pose as two steps. First, we predict robot pose ${\text{ξ}}_{t+1}$ and get the state vector that includes the new camera pose, ${\hat{s}}_{t+1}={\left[{\text{ξ}}_{t},{\text{ξ}}_{t+1},M\right]}^{T}$. Second, we marginalizing ${\text{ξ}}_{t}$ from the posterior to achieve the desired distribution $s_{t+1}={\left[{\text{ξ}}_{t+1}M\right]}^{T}$. This is exemplified in Figure 2.

In this work we estimate the camera motion between RGB-D images through the visual odometry algorithm, explained in detail in Section 6. The observation model, based on 3D landmarks with binary descriptors, will be shown in Section 5.

## 4. RGB-D Image Feature Descriptor

There are many feature descriptor methods, which are divided into two categories: gradient histogram-based feature descriptors, like SIFT [41] and SURF [42], and binary feature descriptors, like BRISK [13], ORB (Oriented Fast and Rotated BRIEF) [43], BRIEF (Binary Robust Independent Elementary Features) [14]. The evaluation criteria of the descriptor include invariance to image noise, scale, translation and rotation transformations. The traditional SIFT and SURF methods are very robust, but the computation time is not practicable for real-time scenes. Binary feature descriptors are described with a binary string. These descriptors are computed by pairwise intensity comparison tests, using simple intensity difference tests, which have the characteristics of less memory consumption, faster processing in creation and a matching process. The distance between two binary strings can be measured using the Hamming distance. The Hamming distance equation is given in equation:

(8) $${\text{Δ}}_{hamming}\left(x,y\right)=\sum_{i=1}^{n}x_{i}⊕y_{i}=\sum_{i=1}^{n}b(x_{i},y_{i})$$

where b(x,y) represents bit inequality and $x_{i}$ and $y_{i}$ are the *i*-th bits in the descriptors *x* and *y*, respectively.

(9) $$b\left(x,y\right)=\left\{\begin{array}{c} 1x\neq y\\ 0x=y \end{array}\right.$$

In this work, we adopt BRAND [12], which combines appearance and geometric shape information from RGB-D images. Compared to other descriptors based on texture, geometry and a combination of both pieces of information, BRAND has advantages in accuracy, processing time and memory consumption, since it combines intensity and geometric information to improve the ability of fast and accurate matching. It is invariant to rotation and scale transform and suitable for applications with low memory consumption and high speed. The algorithm is composed of three main steps:

**Step 1.**  We use the depth information from the RGB-D image to compute the scale factor, which is used in Step 2, and analysis the feature in the keypoint’s neighborhood.
**Step 2.**  We extract a patch in the RGB domain to estimate the feature angular direction of the keypoint’s vicinity.
**Step 3.**  We combine both appearance and geometric information to bring forth keypoint descriptors with a binary string.

**Figure 3:**
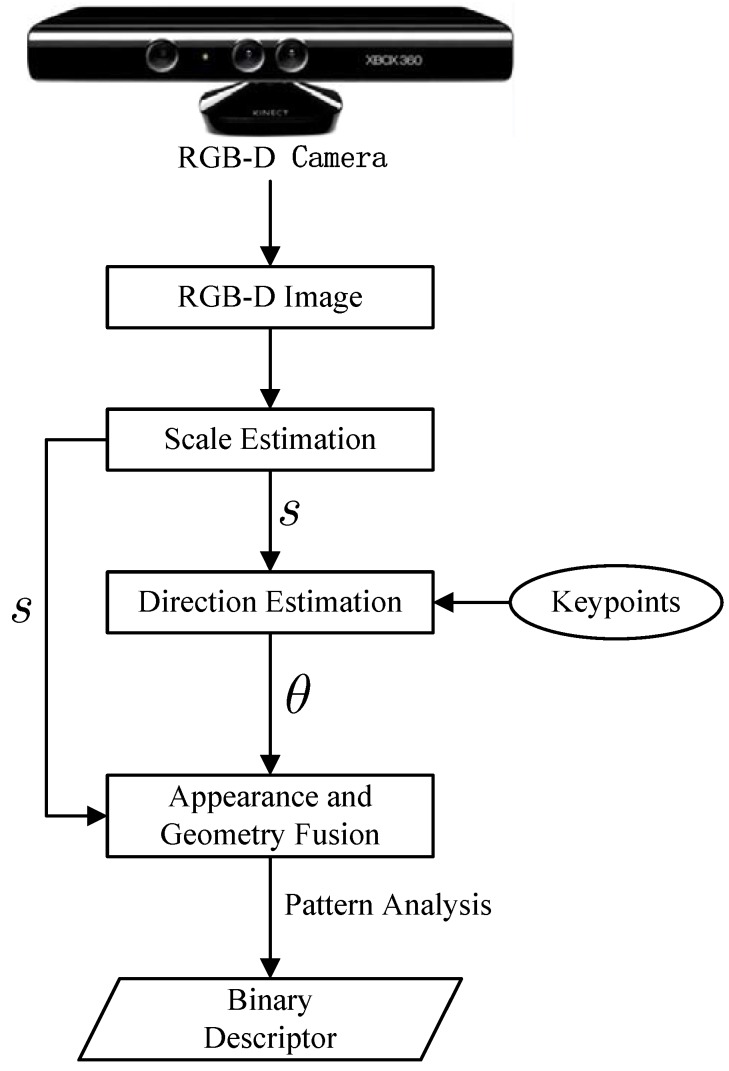
Flow diagram of binary robust appearance and normals descriptor (BRAND) descriptor. *s* is the scale factor, and *θ* is the dominant direction of the keypoint.

**Figure 4:**
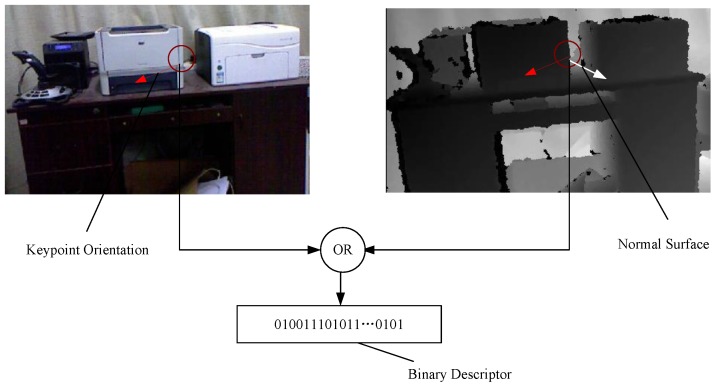
The construction process of the bit string. The red circle represents the patch of size W×W(9≤W≤48) centered at the keypoint location. For sampled pair (x,y) in a patch *P* , the changes in the intensity and geometry are evaluated.

The steps performed to build the binary string are illustrated in Figure 3. The pair (I,D) represents the output of an RGB-D system, where I(x) and D(x) denote color and depth information of a pixel **x**. In the BRAND algorithm, each pair $(x_{i},y_{i})\in P$ is evaluated:

(10) $$f\left(x_{i},y_{i}\right)=\left\{\begin{array}{c} 1\;\;if\;\left(p_{i}\left(x_{i}\right)<p_{i}\left(y_{i}\right)\right)\vee \tau_{g}\left(x_{i},y_{i}\right)\\ 0\;\;otherwise \end{array}\right.$$

where $p_{i}\left(x\right)$ denotes the pixel intensity of a pixel **x** and $p_{i}\left(x_{i}\right)<p_{i}\left(y_{i}\right)$ represents the characteristic gradient changes in the keypoint neighborhood. $\tau_{g}\left(x_{i},y_{i}\right)$ evaluates the geometric pattern on its surface. The analysis of the geometric pattern using τ(.) is based on two invariant geometric measurements: the normal displacement and the surface’s convexity. Figure 4 shows the construction process of the bit string.

We evaluate changes in the intensity and geometry of the sampled pair (x,y) in a patch *p* and encode the descriptor extracted from a patch *p* associated with a keypoint **k**. It is represented as a binary string:

(11) $$b\left(k\right)=\sum_{i=1}^{256}2^{i-1}f\left(x_{i},y_{i}\right)$$

The BRAND descriptor takes into account appearance and geometry from RGB-D images. Appearance is an object property invariant to any geometric transformation, and geometric measurements are invariant to rotation, translation and scaling. Different from descriptors that use either appearance information or geometric information, the BRAND descriptor spends little memory space and little processing time without losing accuracy, which presents invariance to rotation, translation, scale transform and robustness to different illumination conditions.

## 5. RGB-D Feature Observation Model

### 5.1. The Overall RGB-D Observation Model

The probabilistic observation model is $p(z_{t}|{\text{ξ}}_{t})$. Assuming conditional independency in the observation of the individual landmark $z_{t}^{i}$ of the same frame, we get $p\left(z_{t}|{\text{ξ}}_{t}\right)=\prod_{i}p(z_{t}^{i}|{\text{ξ}}_{t})$. In order to avoid explicit data correlation between features in the current frame and map, we employ the full probability method to calculate the marginal distribution of the observation likelihood with individual landmark:

(12) $$p\left(z_{t}^{i}|{\text{ξ}}_{t}\right)=\prod_{\psi =1,2,\cdots ,M,\varphi }p(z_{t}^{i}|{\text{ξ}}_{t},k_{i}=\psi )P\left(k_{i}=\psi |{\text{ξ}}_{t}\right)$$

where $k_{i}$ is a discrete variable, which represents the correspondence of the *i*-th observed landmark. $k_{i}=\varphi$ indicates no correspondence. Since $P(k_{i}=\psi |{\text{ξ}}_{t})$ relies on the observation $z_{t}^{i}$, we assume $P(k_{i}=\psi |{\text{ξ}}_{t})=\varsigma$, where *ς* is constant. Equation (12) is equivalent to $p\left(z_{t}^{i}|{\text{ξ}}_{t}\right)=\varsigma \prod_{\psi =1,2,\cdots ,M,\varphi }p(z_{t}^{i}|{\text{ξ}}_{t},k_{i}=\psi )$, where $p(z_{t}^{i}|{\text{ξ}}_{t},k_{i}=\psi )$ is the probability of the observed landmark $z_{t}^{i}$ and its corresponding landmark $m_{\psi }$ to coincide in both the 3D space of the position and the space of the brand descriptors. It is represented as a Gaussian distribution:

(13) $$\begin{array}{ccc} & & p\left(z_{t}^{i}|{\text{ξ}}_{t},k_{i}=\psi \right)=N\left(0;\underset{\mu }{\underset{︸}{{\bar{z}}_{t}^{i}-{\bar{m}}_{\psi }}},\underset{\text{Σ}}{\underset{︸}{{\text{Σ}}_{z_{t}^{i}}+{\text{Σ}}_{m_{\psi }}}}\right)\\ & = & \varsigma^{'}\exp\left\{-\frac{1}{2}{\left({\bar{z}}_{t}^{i}-{\bar{m}}_{\psi }\right)}^{T}{\left({\text{Σ}}_{z_{t}^{i}}+{\text{Σ}}_{m_{\psi }}\right)}^{-1}\left({\bar{z}}_{t}^{i}-{\bar{m}}_{\psi }\right)\right\} \end{array}$$

where $\varsigma^{'}={\left(2\pi \left|{\text{Σ}}_{z_{t}^{i}}+{\text{Σ}}_{m_{\psi }}\right|\right)}^{-\frac{1}{2}}$.

The exponential term in Equation (13) can be divided into two factors related to the position and descriptor dimensions of the random variable separately:

(14) $$\begin{array}{cc} & N\left(0;\mu ,\text{Σ}\right)\\ = & \varsigma^{'}\exp\left\{-\frac{1}{2}\left(\mu_{p}^{T}\;\mu_{F}^{T}\right){\left(\begin{array}{cc} {\text{Σ}}_{p} & 0\\ 0^{T} & {\text{Σ}}_{F} \end{array}\right)}^{-1}\left(\begin{array}{c} \mu_{p}^{}\\ \mu_{F}^{} \end{array}\right)\right\}\\ = & \varsigma^{'}\exp\left\{-\frac{1}{2}\left(\mu_{p}^{T}{{\text{Σ}}_{p}}^{-1}\mu_{p}^{}+\mu_{F}^{T}{{\text{Σ}}_{F}}^{-1}\mu_{F}^{}\right)\right\}\\ = & \varsigma^{'}\exp\left\{-\frac{1}{2}\mu_{p}^{T}{{\text{Σ}}_{p}}^{-1}\mu_{p}^{}\right\}\exp\left\{-\frac{1}{2}\mu_{F}^{T}{{\text{Σ}}_{F}}^{-1}\mu_{F}^{}\right\} \end{array}$$

where ${\text{Σ}}_{p}$ and ${\text{Σ}}_{F}$ are related to the position and the descriptor dimensions, respectively. The latter part of Equation (14) is taken for the Euclidean distance between feature descriptors. If the Euclidean distance is used to measure data correlation between features, the data association problem can be resolved well and be integrated into the observation model. However, in challenging scenarios, such as fast camera motions and environments with many similar objects, the feature matching speed is very low. Therefore, we adopt the binary feature descriptor and computer Hamming distance in order to improve the speed of feature matching and to reduce the size of the feature description database.

### 5.2. RGB-D Correlation Model of Observation

In this work, we assume that the *i*-th feature is described as a vector $f_{i}={\left(p_{i},{\text{Σ}}_{i},b_{i}\right)}^{T}$. $p_{i}={\left(x_{i},y_{i},z_{i}\right)}^{T}$ is the 3D location of feature *i* in the world coordinate system; ${\text{Σ}}_{i}$ is the 3×3 covariance matrix of $p_{i}$; and $b_{i}$ is binary description of feature *i*. The observation $z_{t}$ is:

(15) $$z_{t}=\left\{z_{t}^{i}|i=1,2,\cdots ,N_{t}\right\}wherez_{t}^{i}=\langle p_{t}^{i},f_{t}^{i}\rangle$$

where $p_{t}^{i}$ is the 3D location of the i-th landmark in the camera coordinate system at time step *t* and $f_{t}^{i}$ is the feature descriptor of the landmark. Assuming that position uncertainty in 3D is represented as mean *μ* and 3×3 covariance matrix *Σ*:

(16) $$p_{t}^{i}∼N\left(\mu_{t}^{i},{\text{Σ}}_{t}^{i}\right)$$

The map *m* is defined as $\left\{m^{j}|j=1,2,\cdots ,M\right\}$, where $m^{j}=\langle p_{m}^{j},f_{m}^{j}\rangle$, $p_{m}^{j}$ is the 3D location of the *j*-th landmark in the camera coordinate system and $f_{m}^{j}$ is the feature descriptor of the landmark. Assuming the position in the map is a normal distribution:

(17) $$p_{m}^{j}∼N\left(\mu_{m}^{j},{\text{Σ}}_{m}^{j}\right)$$

All bits of the binary descriptor are independent and identically distributed binary random variables, *i.e*., p(0)=p(1)≈0.5. The binary descriptor **F** is:

(18) $$F∼\langle \underset{\text{the first bit}}{\underset{︸}{B(1,0.5)}},\cdots ,\underset{\text{the L-th bit}}{\underset{︸}{B(1,0.5)}}\rangle$$

Every bit value of the feature descriptor is not important, but we need to know the similarity between the two feature descriptors, *i.e*., the distribution of the Hamming distance $H=\left|f^{i},f^{j}\right|$.

Let $l_{k}$ is the probability of a successful match for the *k*-th bit; L is the length of the descriptor. Obviously, $l_{k}$ obeys the binomial distribution, and for the large sample data, the expectations of $l_{k}$ are equal to 0.5. Therefore, the Hamming distance *H* is the sum of *L* variables that obey identical binomial distribution B(1,0.5), and *H* obeys binomial distribution B(L,0.5).

(19) H∼B(L,0.5)

In order to describe the uncertainty of data correlation and geometric measurement in the observation model, we approximate the binomial distribution as the following normal distribution.

(20) H∼N(0.5L,0.25L)

That is, ${\text{Σ}}_{F}$ in Equation (14) is set as 0.25L.

## 6. Motion Model: Dense Visual Odometry

Visual odometry [37,44] is an estimation process of the movement information of an intelligent body only using the input information of a single or multiple cameras. In this paper, we use the DVO proposed by Kerl *et al.* [37] to estimate the ego-motion of RGB-D sensor, which is used as the motion model of EIF. DVO estimates camera motion by aligning two consecutive RGB-D images.

A 3D point *p* in the scene observed by two cameras is assumed to yield the same brightness in both images, *i.e*., $I_{1}\left(x\right)=I_{2}\left(\tau \left(u,x\right)\right)$. This assumption is based on the photo-consistency theory. τ(u,x) is the warping function; $u\in R^{6}$ is the camera motion; τ(u,x) maps a pixel coordinate $x\in R^{2}$ in the first image ($I_{1})$ to a coordinate in the second image($I_{2})$. In the following, we will give a detailed derivation of the warping function, calculate the error function based on all of the pixels and minimize the difference between the estimated and the real depth measurements.

### 6.1. Camera Model

We reconstruct a 3D point *p* from its pixel coordinates $x={\left(u_{x},v_{x}\right)}^{T}$ and a corresponding depth measurement ${\text{D}}_{1}\left(x\right)$ using the inverse projection function $\pi^{-1}$, *i.e.*,

(21) $$\begin{array}{c} p=\pi^{-1}\left({x,\text{D}}_{1}\left(x\right)\right)\\ ={\text{D}}_{1}\left(x\right){\left(\frac{u_{x}+u_{0}}{\alpha },\frac{v_{x}+v_{0}}{\beta },1\right)}^{T} \end{array}$$

where *α*, *β* are the focal length and $u_{0}$, $v_{0}$ are the center coordinates of the pinhole camera model.

### 6.2. Warping Function

In the coordinate frame of the second camera, the point **p** is rotated and translated according to the rigid body motion *g* (g∈SE(3), which is the special Euclidean group). A rigid body motion comprises a rotation matrix **R** (R∈SO(3), which is the rotation group) and a translation vector **t** ($t\in R^{3}$). The transformation matrix **T** is given as:

(22) $$T_{4\times 4}=\left[\begin{array}{cc} R_{3\times 3} & t_{3\times 1}\\ 0 & 1 \end{array}\right]$$

The transformation of the 3D point **p** with *g* is g(p)=Tp. **T** has twelve parameters, while *g* has six degrees of freedom. Therefore, we use twist coordinates **u**; **u** is a six-vector, *i.e*., $u={\left(v_{1},v_{2},v_{3},w_{1},w_{2},w_{3}\right)}^{T}$. $v_{1},v_{2},v_{3}$ are called the linear velocity and $w_{1},w_{2},w_{3}$ are the angular velocity of the motion. The transformation matrix **T** can be calculated from **u** using the matrix exponential $T=\exp(\hat{u})$ relating Lie algebra se(3) to Lie group SE(3).

When the transformed point $T\left(p\right)={\left(x,y,z\right)}^{T}$ is observed by the second camera, we calculate warped pixel coordinates as:

(23) $$\pi \left(T\left(p\right)\right)={\left(\frac{\alpha x}{z}-u_{0},\frac{\beta y}{z}-v_{0}\right)}^{T}$$

We summarize Equation (21)–(23); the full warping function is:

(24) $$\begin{array}{c} \tau (x,T)=\pi (Tp)\\ =\pi (T\left(\pi^{-1}\left({x,D}_{1}\left(x\right)\right)\right) \end{array}$$

### 6.3. Probabilistic Estimation

The difference in brightness between the first and the warped second image is defined as:

(25) $$r_{i}\left(u\right)=I_{2}\left(\tau \left(x_{i},T\right)\right)-I_{1}\left(x_{i}\right)$$

By assuming that all *n* pixels $x_{i}$ (i=1,⋯,n) in the image are equal, the probability of whole residual image $r={\left(r_{1},\cdots ,r_{n}\right)}^{T}$ is $p\left(r|u\right)=\prod_{i}p\left(r_{i}|u\right)$. After applying Bayes’ rule, the posterior probability of a camera motion *u* given a residual image *r* is:

(26) $$p\left(u|r\right)=\frac{p(r|u)p(u)}{p(r)}$$

We seek for $u_{MAP}$ by maximizing the posterior probability, *i.e*.,

(27) $$u_{MAP}=\arg\max_{u}p\left(u|r\right)$$

By integrating Equation (26) with Equation (27) and removing the term p(r), which does not depend on *u*, we obtain:

(28) $$u_{MAP}=\arg\max_{u}\prod_{i}p\left(r_{i}|u\right)p\left(u\right)$$

Assuming all residuals $r_{i}$ (i=1,⋯,n) are independent and identically distributed, by minimizing instead the negative log likelihood, we get:

(29) $$u_{MAP}=\arg\min_{u}-\sum_{i}\log p\left(r_{i}|u\right)-\log p\left(u\right)$$

The minimum is found when the derivative of the log likelihood is set to zero. To simplify Equation (29), we drop the motion prior logp(u) and obtain:

(30) $$\sum_{i}\frac{\partial \log p(r_{i}|u)}{\partial u}=\sum_{i}\frac{\partial \log p(r_{i})}{\partial r_{i}}\frac{\partial r_{i}}{\partial u}=0$$

We define $w\left(r_{i}\right)=\partial \log p\left(r_{i}\right)/\partial r_{i}\cdot 1/r_{i}$ and get $\frac{\partial r_{i}}{\partial u}w\left(r_{i}\right)r_{i}=0$. The photometric error follows a t-distribution [45] $p_{t}\left(0,\sigma^{2},v\right)$. In the distribution, mean μ=0, and variance =$\sigma^{2}$; degree of freedom = *v*. In Equation (29), we assume that all residuals are equal. It is very difficult to satisfy. Really, large errors covering the outliers get low weights. On the contrary, small errors with large variance get higher probability. The t-distribution is fit for this model. If $p(r_{i}|u)$ is defined as a t-distribution, the weighted least squares problem is:

(31) $$u_{MAP}=\arg\min_{u}\sum_{i}{w\left(r_{i}\right)\left(r_{i}\left(u\right)\right)}^{2}$$

The function $w(r_{i})$ is called the weighting function and is defined as:

(32) $$w\left(r_{i}\right)=\partial \log p\left(r_{i}\right)/\partial r_{i}\cdot 1/r_{i}$$

In this paper, the residual $r_{i}$ follows a bivariate t-distribution. Based on the t-distribution $p_{t}\left(0,\text{Σ},v\right)$, the weights $w(r_{i})$ are:

(33) $$w\left(r_{i}\right)=\frac{v+1}{v+r_{i}^{T}\sum^{-1}r_{i}}$$

The weight $w(r_{i})$ termed by ∑ is automatically adapted.

### 6.4. Optimization of Motion Estimation

This optimization problem is a non-linear least squares problem. The residuals $r_{i}\left(u\right)$ are non-linear in *u*; we use a first order Taylor expansion to linearize it. We obtain normal equations of this non-linear least squares problem:

(34) $$\begin{array}{c} A\text{Δ}u=b\\ \sum_{i}^{n}w_{i}J_{i}^{T}\sum^{-1}J_{i}\text{Δ}u=-\sum_{i}^{n}w_{i}J_{i}^{T}{\text{Σ}}^{-1}r_{i} \end{array}$$

where $J_{i}\in R^{2\times 6}$ is the Jacobian matrix, which contains the derivatives of $r_{i}$ concerning *u*. The normal equation for increments Δu is iteratively calculated. At each iteration, the scale matrix ∑ and the weights $w_{i}$ are re-estimated. A is the Hessian matrix of nonlinear least squares problems. Assuming parameters *u* are normally distributed, $A^{-1}$ is a lower bound for the variance of the estimated parameters *u*, *i.e*., $\sum_{u}=A^{-1}$.

## 7. Experimental Results

The experiments were implemented with the Robot Operating System (ROS) framework. All of the experiments were done using the same notebook computer, which has an Intel Core i7-4700HQ CPU and 8.0 GB RAM with Ubuntu 14.04 64-bit operation system. In the experiments, we compared three methods: the VO-EIF RGB-D SLAM, the DVO algorithm and a type of graph-based RGB-D SLAM implemented in the RTAB-Map system [46]. In the graph-based RGB-D SLAM, the TORO (tree-based network optimizer) [47] is selected to optimize the pose graph, and FAST/BRAND is selected as the detector/descriptor [48]. The other parameter settings of the graph-based RGB-D SLAM are the same as in [48], except that time limit *T* is not set. In our VO-EIF RGB-D SLAM, we set two update thresholds $t_{1}$ and $t_{2}$. When any of the following two conditions are satisfied, the filter update will execute: the accumulated changes of *x*, *y* or *z* of the visual odometry are greater than $t_{1}$, or any of the accumulated changes of roll, pitch or yaw of the visual odometry are greater than $t_{2}$. In the following experiments, $t_{1}$ is set to 0.1 m and $t_{2}$ is set to 0.1 rad.

In order to test the validity of our algorithm, we implemented two different experiments. The first experiment was conducted online in our lab environment, which focused on the effectiveness and timeliness of all parts of our algorithm, as well as qualitatively verifying the accuracy. The second was accomplished off-line by utilizing the RGB-D benchmark provided by the Technical University of Munich [31]. The advantage of using this benchmark is that each dataset of the benchmark accompanies an accurate ground truth trajectory obtained with an external motion capture system, and it can be used to quantitatively evaluate the accuracy of the algorithm. Benchmark data were taken with a Microsoft Kinect sensor, providing 640×480 RGB and depth frames at a 30-Hz rate; the ground truth data were taken with a highly accurate motion capture system, composed of eight 100-Hz cameras.

### 7.1. Lab Environment Results

**Figure 5:**
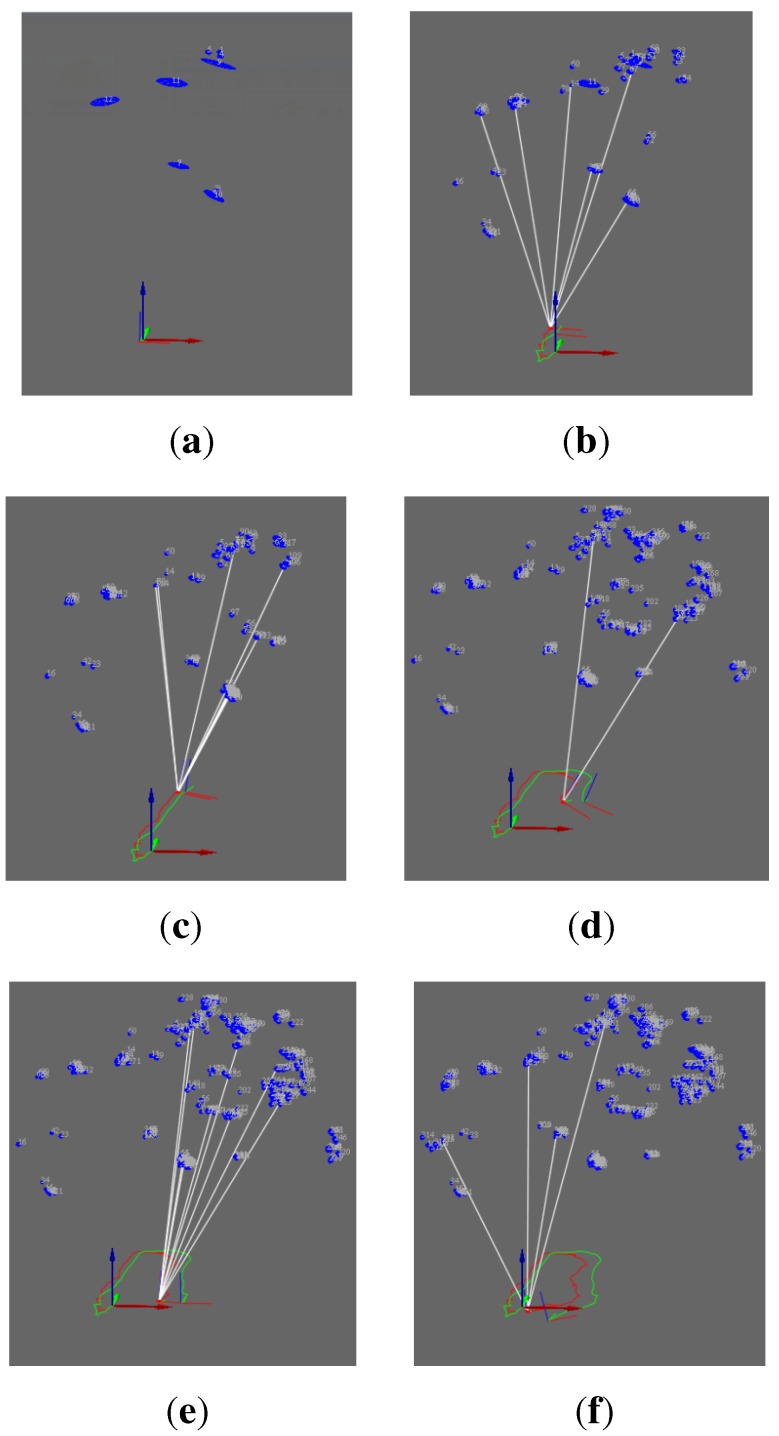
The map building process of VO-EIF RGB-D SLAM: (**a**) The first frame; (**b**) The eighth frame; (**c**) The thirty-first frame; (**d**) The fifty-second frame; (**e**) The sixty-first frame; (**f**) The seventy-third frame.

Figure 5 shows landmark observation in the process of the camera motion. Figure 5a demonstrates the observation of the first frame. Seen from the view of xz (the red arrow represents the *x*-axis and the blue arrow represents the *z*-axis), no landmark is observed in the first frame (the blue oval represents the landmark). In Figure 5b–f, white lines represent landmark re-observation. The number of keypoints for observation is limited to 10 for the purpose of improving the speed of SLAM. From Figure 5b–f, we can see that the landmarks number gradually increased and the uncertainties of re-observed landmarks gradually decreased.

**Figure 6:**
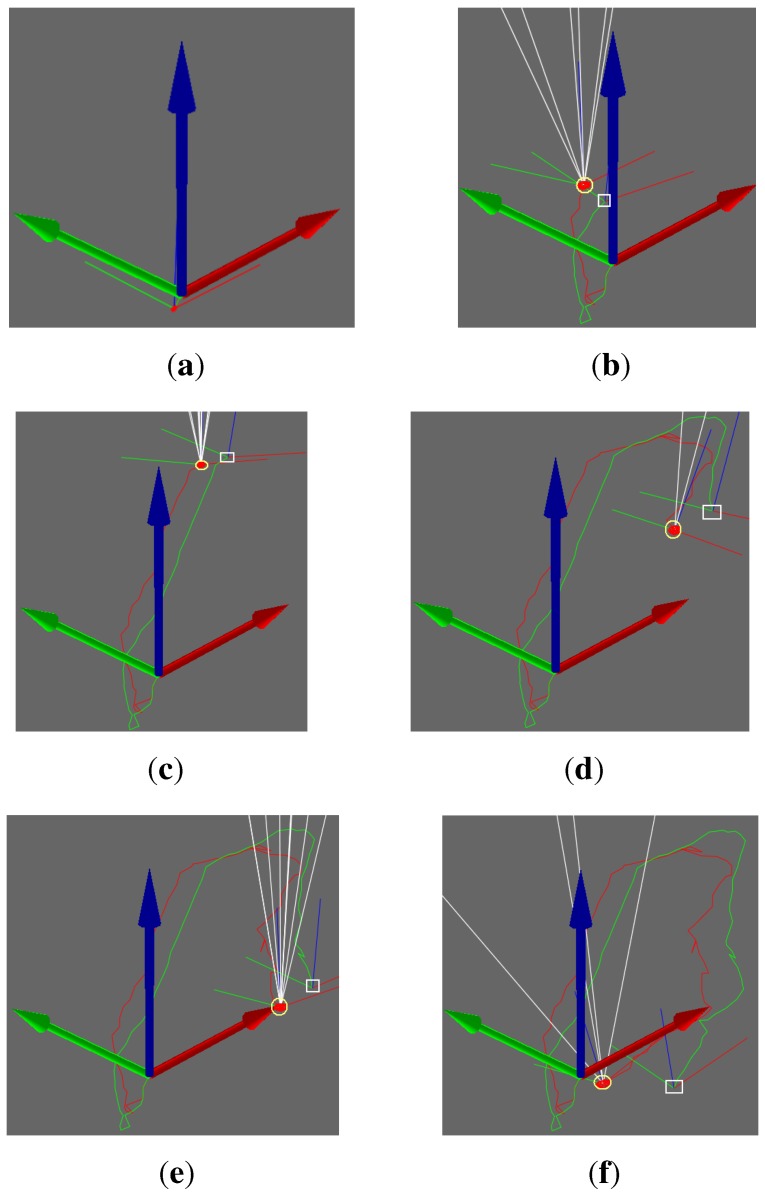
Comparison of two trajectories: (**a**) The first frame; (**b**) The eighth frame; (**c**) The thirty-first frame; (**d**) The fifty-second frame; (**e**) The sixty-first frame; (**f**) The seventy-third frame.

Figure 6 demonstrates a comparison between two trajectories of the two methods. The red curve stands for the motion trajectory with the VO-EIF RGB-D SLAM algorithm, and the green curve stands for the motion trajectory with traditional visual odometry. In Figure 6b–f, the yellow circles represent the camera position estimated with the VO-EIF RGB-D SLAM method, and the white square represents the camera position estimated with the DVO method. Seen from Figure 6, the difference between the two methods is not obvious in the first 30 frames of the motion. In the later stage of the motion, when the camera moves toward the original position, the red trajectory is close to the original position, but the green trajectory deviates from the original position. Without the observation model, accumulative error with the DVO method increased with time and affected proper trajectory estimation. In each step of the observation of the VO-EIF RGB-D SLAM method, the error is very small, and accumulative error can be corrected. Therefore, the algorithm of VO-EIF RGB-D SLAM can properly estimate camera motion.

These results show that VO-EIF RGB-D SLAM has the advantage of smaller accumulative error. We acquire the trajectory closer to the real trajectory with the VO-EIF RGB-D SLAM. Especially, when the camera comes back to the original position, the trajectory with the traditional method deviates from the original position, but the trajectory with the VO-EIF RGB-D SLAM is very close to the original position (as can bee seen in Figure 6f).

### 7.2. Benchmark Results

The results of the benchmark experiments were calculated using the absolute trajectory error (ATE) evaluation tool provided with the benchmark. This evaluation method directly compares the difference between poses in the ground truth and measured trajectory, and the end result of it is the root mean squared error (RMSE) of the per pose errors summed over the entire trajectory.

**Figure 7:**
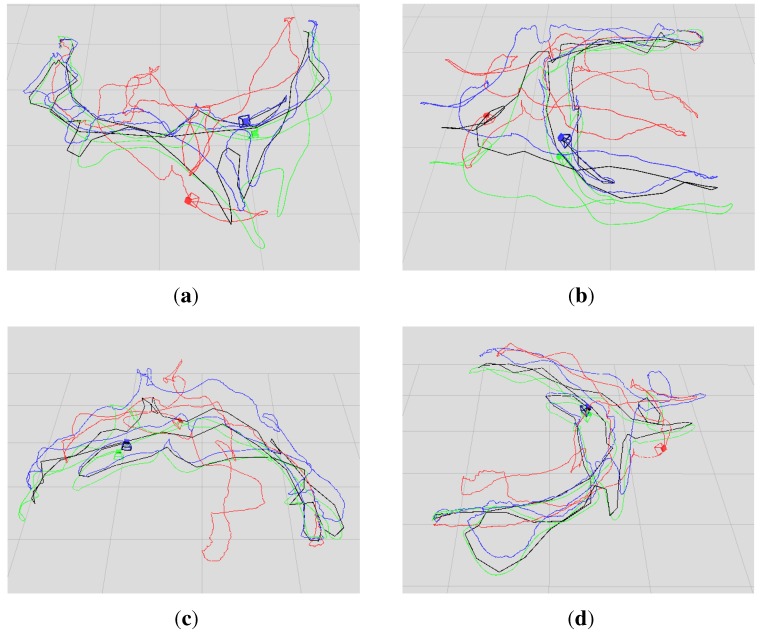
Comparison of the four trajectories for the sequence “freiburg1_room”. The blue, red and black trajectories are generated by our algorithm, the DVO algorithm and the graph-based algorithm, respectively. The green trajectory is the ground truth. (**a**–**d**) The views in four different perspectives.

**Figure 8:**
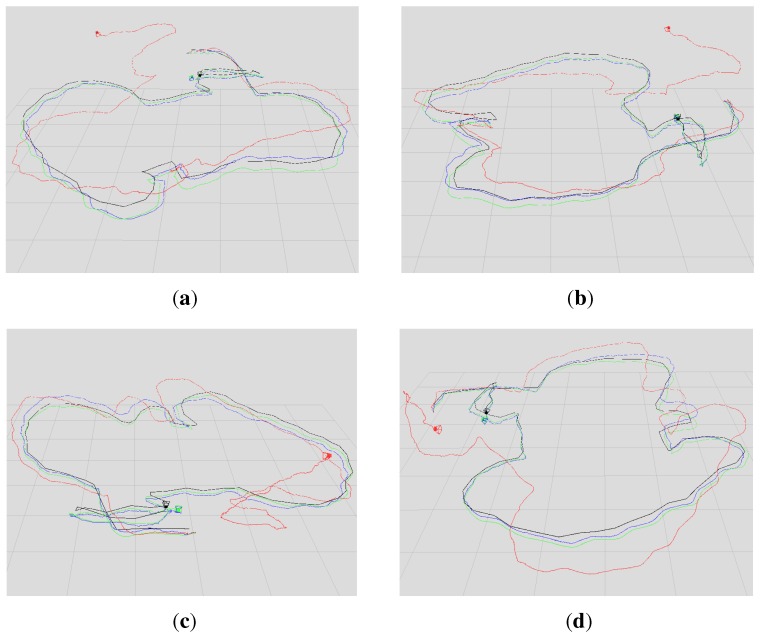
Comparison of the four trajectories for the sequence “freiburg3_long_office_household”. The blue, red and black trajectories are generated by our algorithm, the DVO algorithm and the graph-based algorithm, respectively. The green trajectory is the ground truth. (**a**–**d**) The views in four different perspectives.

**Figure 9:**
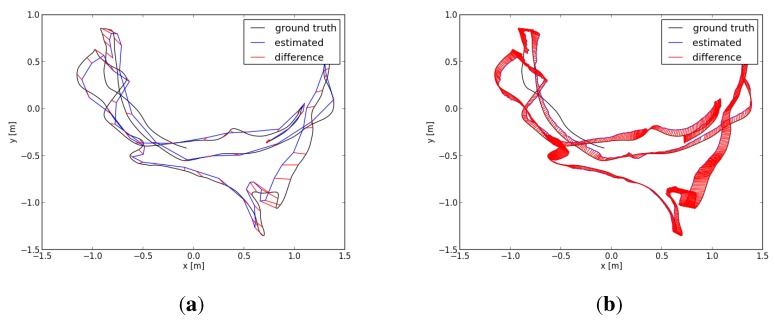
Comparing absolute trajectory errors (ATEs) for the sequence “freiburg_1room”: (**a**) ATE using the graph-based RGB-D SLAM; (**b**) ATE using the VO-EIF RGB-D SLAM.

**Figure 10:**
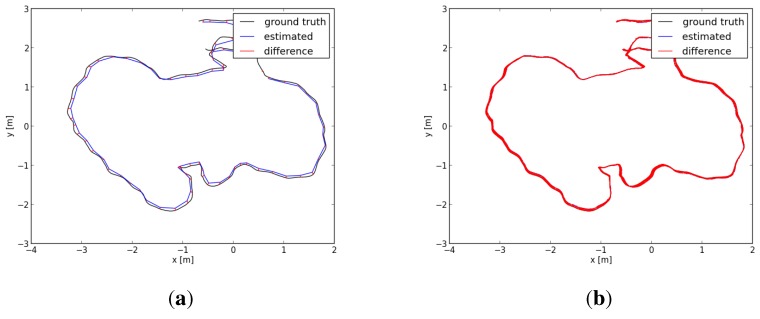
Comparing ATEs for the sequence “freiburg3_long_office_household”: (**a**) ATE using the graph-based RGB-D SLAM; (**b**) ATE using the VO-EIF RGB-D SLAM.

In this paper, we evaluated two datasets using our proposed algorithm, the DVO algorithm and the above graph-based RGB-D SLAM separately. The two datasets are sequences “freiburg1_room” and “freiburg3_long_office_household”. Their durations are 48.90 s and 87.09 s, respectively. The statistical results are shown in Table 1 and Table 2. Figure 7 shows in four different perspectives the trajectory results of the sequence “freiburg1_room”: the ground truth trajectory and the three trajectories, which are respectively generated by the three algorithms. Similar to Figure 7, Figure 8 is the trajectory results of the sequence “freiburg3_long_office_household”. The measured trajectory errors are shown in Figure 9 and Figure 10. From these experimental results, we can see that the VO-EIF RGB-D SLAM can successfully complete the large loop closing, while the DVO cannot (as can be seen in Figure 10a). This is because in VO-EIF RGB-D SLAM, the re-observed features can greatly improve the sensor localization accuracy. The localization precision of our algorithm is nearly the equivalent of the graph-based algorithm. It should be pointed out that the trajectory of the graph-based algorithm is nearly fully updated at every update time, but in our filter-based algorithm, only the current camera pose is updated, and the poses of the passed time are not saved and updated in the filter. In other words, in the filter-based algorithm, the estimation of the camera pose at time *t* is only based on the information by time *t*, which has no post updating.

**Table 1:** Comparison results of processing the sequence “freiburg1_room”. DVO, dense visual odometry.

Comparison Index Terms		Method		
		DVO	VO-EIF RGB-D SLAM	Graph RGB-D SLAM
	**RMSE**	0.447535	0.114760	0.093479
**Trajectory**	**Mean**	0.418295	0.109373	0.083075
**error**	**Median**	0.412622	0.112226	0.072460
**indicators**	**STD**	0.159112	0.034747	0.042859
**(m)**	**Min**	0.092879	0.024764	0.025987
	**Max**	0.794312	0.309430	0.211681
**Total processing time (s)**		15.7327	24.6747	105.8131

**Table 2:** Comparison results of processing the sequence “freiburg3_long_office_household”.

Comparison Index Terms		Method		
		DVO	VO-EIF RGB-D SLAM	Graph RGB-D SLAM
	**RMSE**	0.535152	0.067632	0.053803
**Trajectory**	**Mean**	0.428854	0.061255	0.051310
**error**	**Median**	0.271139	0.053549	0.050743
**indicators**	**STD**	0.320113	0.028669	0.016189
**(m)**	**Min**	0.090521	0.010577	0.023585
	**Max**	1.124703	0.130501	0.124373
**Total processing time (s)**		29.9347	63.4500	184.4862

**Figure 11:**
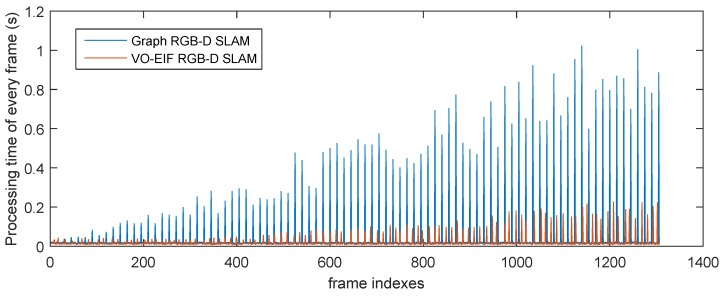
Total processing time of every frame of the sequence “freiburg1_room” by different algorithms.

**Figure 12:**
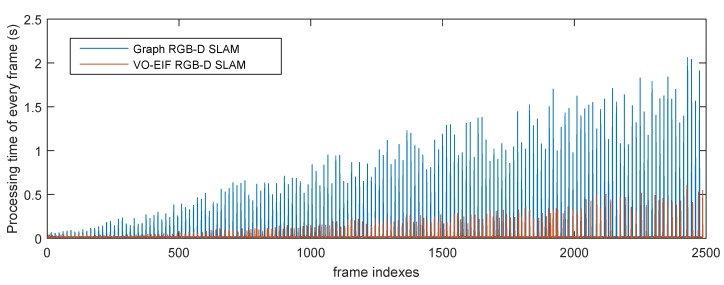
Total processing time of every frame of the sequence “freiburg3_long_office_household” by different algorithms.

Figure 11 and Figure 12 show the processing time for each frame of the two sequences by different algorithms. It can be seen that the update time of our algorithm is smaller than the processing time of the graph-based algorithm for the keyframe at a similar moment.

## 8. Conclusions

In this paper, we put forward a novel RGB-D SLAM algorithm. Our RGB-D observation model is based on the binary descriptor, which effectively reduces the time complexity of the data association. Visual odometry is estimated for the movement of the camera by aligning two consecutive intensity images $I_{1}$ and $I_{2}$ and incorporating the weight and motion prior. We evaluated our approach quantitatively on a publicly-available RGB-D dataset and compare our approach to a graph-based RGB-D SLAM algorithm. The experimental results illustrate that VO-EIF RGB-D SLAM can successfully complete large loop closing, and the localization precision of our algorithm is nearly the equivalent of the graph-based algorithm; moreover, our algorithm is quicker than the graph-based algorithm.

As a next step, we plan to extend the VO-EIF RGB-D SLAM algorithm to the map with a 3D color point cloud. The VO-EIF RGB-D SLAM algorithm based on the keyframe [32] will be studied. For example, if there are many (at least three) feature points in the observation of the camera at time *t*, the frame can be used as a candidate keyframe. After the corresponding landmark of the features is updated, the pose of the keyframe is updated by reverse-measurement. Furthermore, a more sophisticated technique could be used to extract image features for a more robust and efficient system.
